# Combined Treatment (Ultraviolet-C/Physapruin A) Enhances Antiproliferation and Oxidative-Stress-Associated Mechanism in Oral Cancer Cells

**DOI:** 10.3390/antiox11112227

**Published:** 2022-11-11

**Authors:** Sheng-Yao Peng, Ching-Yu Yen, Ting-Hsun Lan, Jiiang-Huei Jeng, Jen-Yang Tang, Hsueh-Wei Chang

**Affiliations:** 1 Department of Biomedical Science and Environmental Biology, Ph.D Program in Life Sciences, College of Life Sciences, Kaohsiung Medical University, Kaohsiung 80708, Taiwan; 2 Department of Oral and Maxillofacial Surgery, Chi-Mei Medical Center, Tainan 71004, Taiwan; 3 School of Dentistry, Taipei Medical University, Taipei 11031, Taiwan; 4 Division of Prosthodontics, Department of Dentistry, Kaohsiung Medical University Hospital, Kaohsiung 80708, Taiwan; 5 School of Dentistry, College of Dental Medicine, Kaohsiung Medical University, Kaohsiung 80708, Taiwan; 6 Department of Dentistry, Kaohsiung Medical University Hospital, Kaohsiung 80708, Taiwan; 7 Department of Dentistry, National Taiwan University Hospital, Taipei 100225, Taiwan; 8 School of Post-Baccalaureate Medicine, Kaohsiung Medical University, Kaohsiung 80708, Taiwan; 9 Department of Radiation Oncology, Kaohsiung Medical University Hospital, Kaohsiung 80708, Taiwan; 10 Center for Cancer Research, Kaohsiung Medical University, Kaohsiung 80708, Taiwan; 11 Institute of Medical Science and Technology, National Sun Yat-sen University, Kaohsiung 80424, Taiwan

**Keywords:** UVC, *Physalis peruviana*, combined treatment, oral cancer, oxidative stress

## Abstract

Physapruin A (PHA), a *Physalis peruviana*-derived withanolide, exhibits antiproliferation activity against oral and breast cancer cells. However, its potential antitumor effects in combined treatments remain unclear. This investigation focused on evaluating the impact of the combined treatment of ultraviolet-C with PHA (UVC/PHA) on the proliferation of oral cancer cells. The UVC-caused antiproliferation was enhanced by combination with PHA in oral cancer (Ca9-22 and CAL 27) but not normal cells (SG), as evidenced by ATP detection, compared with UVC or PHA alone. UVC/PHA showed a greater extent of subG1 increase, G2/M arrest, annexin-V-assessed apoptosis, caspase 3/7 activation, and reactive oxygen species (ROS) in the UVC or PHA treatment of oral cancer compared to normal cells. Moreover, the mitochondrial functions, such as mitochondrial superoxide bursts and mitochondrial membrane potential destruction, of oral cancer cells were also enhanced by UVC/PHA compared to UVC or PHA alone. These oxidative stresses triggered γH2AX and 8-hydroxyl-2’-deoxyguanosine-assessed DNA damage to a greater extent under UVC/PHA treatment than under UVC or PHA treatment alone. The ROS inhibitor *N*-acetylcysteine reversed all these UVC/PHA-promoted changes. In conclusion, UVC/PHA is a promising strategy for decreasing the proliferation of oral cancer cells but shows no inhibitory effect on normal cells.

## 1. Introduction

Oral cancer ranks among the top 10 cancers worldwide [1]. Oral cancer causes hundreds of thousands of deaths every year around the world [2]. Genetic factors interacting with environmental components can individually and collectively influence a person’s susceptibility to cancer, as shown by epidemiological and clinical studies over the past two decades [3]. Surgery, radiation, chemotherapy, and radio-chemotherapy are current oral cancer treatments [4]. Radio-chemotherapy is a treatment that combines anticancer drugs, including radiosensitizers [5], with radiation to improve antiproliferation effects. However, the combined antiproliferation treatment strategy is sometimes ineffective in cancer therapy [6,7,8] due to its adverse effects when combined with radiosensitizers that exhibit cytotoxic effects on normal cells. Consequently, the ideal radiosensitizers are expected to show low toxicity to normal cells and spontaneously improve the radiation-killing outcomes for cancer cells [9].

Several natural products have been reported in combined treatment with radiotherapy [10,11] to alleviate resistance or adverse effects and improve cancer treatment [12,13]. In addition to ionizing radiation (X-rays), non-ionizing radiation such as ultraviolet-C (UVC) also exhibits antiproliferation effects on several cancer types, such as pancreatic [14], colon [15], oral [16], and glioblastoma multiforme [17] cancer cells. UVC was combined with several clinical drugs [18,19] and natural products [16,20] to demonstrate anticancer effects.

*Physalis peruviana* L., a withanolide-rich edible plant [21], is used for traditional medicines in Asia and South America [22]. *P. peruviana* exhibits antiproliferation effects against several cancer types [23,24,25,26]. *P. peruviana*-derived physapruin A (PHA) was reported to be antiproliferative against oral [27] and breast [28] cancer cells. Moreover, PHA exhibits more antiproliferation effects against oral cancer cells than normal cells [27]. This preferential-antiproliferation characteristic of PHA against oral cancer cells provides the benefits of low cytotoxic effects on normal cells and a reduced chance of adverse effects. However, combined treatments using UVC and PHA (UVC/PHA) have rarely been assessed for oral cancer cells.

UVC [29,30] and PHA [27] have been reported to induce oxidative stress, providing a rationale for the proposition that a UVC/PHA combined treatment may cooperatively produce more oxidative stress and antiproliferation than separate treatments (UVC or PHA). Consequently, this investigation aimed to evaluate the enhancement of antiproliferation by UVC/PHA in oral cancer cells. To examine the safety of the treatment, we tested the cytotoxic effects of UVC/PHA on normal oral cells in addition to oral cancer cells.

## 2. Materials and Methods

### 2.1. Cell Culture

Human oral cancer (Ca9-22 and CAL 27) cell lines from JCRB Cell Bank (Osaka, Japan) and ATCC (Manassas, VA, USA) and the non-malignant gingival epithelial Smulow–Glickman (SG) [31,32,33,34] cell line were used. All cell culture media, sera, and antibiotics were obtained from Gibco (Grand Island, NY, USA). Ca9-22 and CAL 27 cells were maintained in Dulbecco’s Modified Eagle Medium (DMEM)/F12 at a 3:2 ratio, and SG cells were kept at a 4:1 ratio [16]. After seeding at 1.5, 1, and 1.5 × 10^5^/6 cm dish for Ca9-22, CAL 27, and SG cells, respectively, the cells were incubated overnight for UVC and/or PHA experiments, as indicated in figure legends.

### 2.2. UVC Irradiation and Drug Treatments

After suctioning out the medium, cells received a UVC (254 nm) irradiation rating of 2 J/m^2^/s [16] for different durations, i.e., 4 s for 8 J/m^2^. The negative control for UVC underwent the same processes but was not treated with UVC.

PHA (BioBioPha Co., Kunming, China) was dissolved in DMSO. Cells were pretreated with *N*-acetylcysteine (NAC) (Sigma-Aldrich, St. Louis, MO, USA) [35,36,37,38] (10 mM, 1 h) to assess the levels of oxidative stress in cells receiving a post-treatment of UVC and/or PHA. The final concentration of DMSO was 0.1% for all experiments with or without PHA. Control (no UVC or PHA), UVC, PHA, and UVC/PHA samples were examined for 24 h of incubation to assess the antiproliferation effects of the combined treatment. UVC and/or PHA treatments were administered at 8 J/m^2^ and 0.8 μM for all cell lines.

### 2.3. Cell Survival and Cell Number Assays and Synergy Determination

Cell survival was evaluated by cellular ATP kits (PerkinElmer Life Sciences, Boston, MA, USA) [16]. One hundred microliters of the cell lysate was reacted with 12.5 μL of D-luciferin and luciferase for 5 min in the dark. Then, the luminescent signaling from the ATP reaction was assessed by a CentroLIApc LB 962 Luminometer (Berthold Technologies GmbH & Co, Bad Wildbad, KG., Germany). Additionally, cell numbers were determined by trypan blue exclusion assay (final concentration 0.2%) [39]. The synergy between UVC and PHA was calculated as previously described [40], i.e., the synergy (α) = survival fraction for UVC × survival fraction for PHA/(survival fraction for UVC/PHA). Values of α > 1, =1, and <1 indicated synergistic, additive, or antagonistic changes, respectively.

### 2.4. Cell Cycle Assays

After 70% ethanol fixation, cellular DNA contents were stained with 7-aminoactinmycin D (7AAD) (Biotium, Hayward, CA, USA) at 5 μg/mL for 30 min [41]. The cell cycle phase was assessed by a Guava easyCyte flow cytometer (Luminex, Austin, TX, USA) and interpreted using FlowJo (Becton-Dickinson, Franklin Lakes, NJ, USA).

### 2.5. Annexin V Assay and Caspase 3/7 (Cas 3/7) Activity

Cells (Ca9-22, CAL 27, and SG) were stained with an annexin V-FITC (1:1000 dilution) (Strong Biotech Corporation, Taipei, Taiwan)/7AAD (1 μg/mL) solution at 37 °C for 30 min [42]. After PBS resuspension, cells were analyzed by a flow cytometer. Cas 3/7 activation is associated with apoptosis signaling, which was detected using a Glo^®^ 3/7 commercial system (Promega; Madison, WI, USA) [16]. A Cas 3/7 substrate (DEVD) was added to the medium at 37 °C for 30 min. Consequently, apoptotic cells containing activated Cas 3/7 could cleave DEVD to generate the luminescent signal detected by the luminometer.

### 2.6. ROS and Mitochondrial Superoxide (MitoSOX) Assays

2’,7’-dichlorodihydrofluorescein diacetate (Sigma-Aldrich), a common ROS reaction probe, was mixed with cells at 10 μM (37 °C, 30 min). This probe became fluorescent and was detected by flow cytometry [43]. Similarly, MitoSOX Red (Invitrogen, Eugene, OR, USA) (50 nM, 37 °C, 30 min) [16] was used for MitoSOX detection using flow cytometry.

### 2.7. Mitochondrial Membrane Potential (MitoMP) Assays

DiOC_2_(3) (Invitrogen) (5 nM, 37 °C, 30 min) [16], a MitoMP probe, can stain high-intensity MitoMP. Once MitoMP is depleted, the MitoMP intensity is low. After PBS resuspension, cells were analyzed by a flow cytometer.

### 2.8. γH2AX and 8-Hydroxyl-2’-Deoxyguanosine (8-OHdG) Assays

γH2AX, a DNA double-strand-break biomarker, was assessed by antibody-related flow cytometry [44]. Fixed cells were incubated with mouse γH2AX antibodies (Santa Cruz Biotechnology, Santa Cruz, CA, USA) (1:500 dilution). Subsequently, they were supplemented with Alexa Fluor 488 secondary antibodies (Cell Signaling Technology, Danvers, MA, USA) (1:10,000 dilution) in the presence of 7AAD (5 μg/mL). After PBS resuspension, cells were analyzed by flow cytometer.

8-OHdG, one of the primary oxidative DNA damage types, was assessed by antibody-related flow cytometry [16]. Fixed cells were incubated with 8-OHdG-FITC antibodies (1:10,000 dilution) (Santa Cruz Biotechnology) (4 °C, 1 h). After PBS resuspension, cells were analyzed by flow cytometer.

### 2.9. Statistics

The significance between multi-comparisons was assessed by one-way ANOVA and Tukey’s HSD post hoc evaluation (JMP12, SAS Institute, Cary, NC, USA). Treatments labeled with different lower-case letters showed significantly different results. All assays were performed in three independent experiments.

## 3. Results

### 3.1. Proliferation-Modulating Effects of UVC/PHA

**Figure 1:**
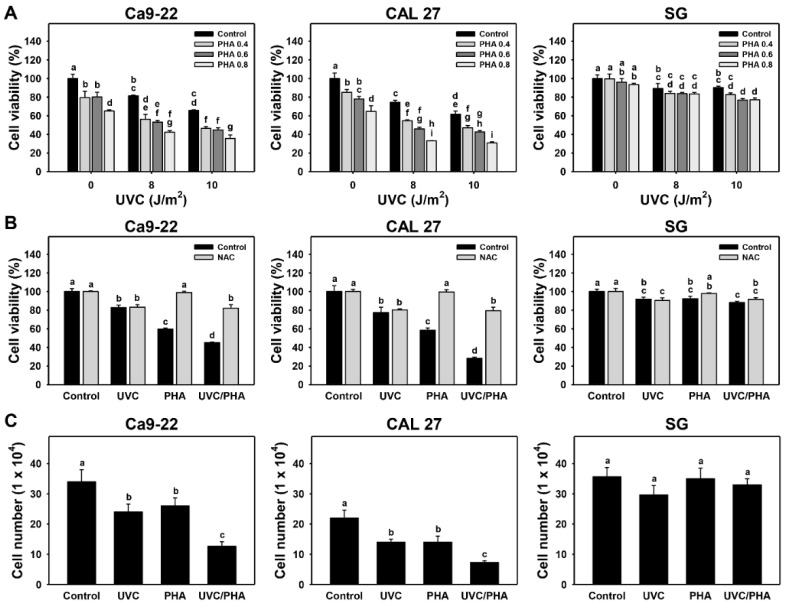
UVC/PHA selectively killed oral cancer cells but not normal oral cells. In the presence or absence of NAC, oral cancer (Ca9-22 and CAL 27) and normal (SG) cells were grouped into four treatments, i.e., control, UVC, PHA, and UVC/PHA, for 24 h. (**A**) ATP assay for different doses of UVC and/or PHA. (**B**) ATP assay for UVC and/or PHA treatments at 8 J/m^2^ and 0.8 μM, respectively. (**C**) Trypan blue exclusion assay. For comparison, treatments labeled with different letters (a to i) produced significantly different results (*p* < 0.05). Data are presented as mean ± SD (*n* = 3 independent experiments, with each experiment performed in three replications).

The cell viability for all tested combined treatments with different UVC and/or PHA doses is shown in Figure 1A. The synergy determinations (α values) for all of these treatments are provided in Supplementary Table S1. The optimal dose for UVC/PHA of 8 J/m^2^, 0.8 μM was chosen for the following experiments. The α values of UVC/PHA (8 J/m^2^, 0.8 μM) in oral cancer cells for the ATP assay (Ca9-22 vs. CAL 27) were 1.15 ± 0.06 and 1.46 ± 0.20, respectively (Figure 1A). Accordingly, the UVC/PHA combined treatment showed a synergistic antiproliferation effect on oral cancer cells.

Oral cancer cells following combined treatment (UVC/PHA) showed a lower cell viability of 45.2% and 28.4% compared to separate treatments (UVC or PHA) in Ca9-22 (82.9% or 59.8%) and CAL 27 (77.5% or 58.4%) cells, respectively, based on the 24 h ATP assay (Figure 1B). In contrast, neither the UVC, PHA, nor UVC/PHA treatments affected cell viability in normal oral SG cells. NAC reversed the UVC/PHA-enhanced antiproliferation, suggesting that UVC/PHA exerted a stronger oxidative-stress-dependent antiproliferation effect on oral cancer cells compared to the separate treatments.

For the trypan blue exclusion assay, UVC/PHA showed lower cell numbers of 37.3% and 33.3% compared to separate treatments (UVC or PHA) in Ca9-22 (70.6% or 76.5%) and CAL 27 (63.6% or 63.6%) cells, respectively (Figure 1C). UVC/PHA produced a mild change in cell numbers (92.5%) compared to the separate treatments (UVC or PHA) in SG cells (83.2% and 98.1%, respectively). Consequently, the effects of UVC/PHA on cell numbers were similar to the results of the ATP assay (Figure 1B).

### 3.2. Cell-Cycle-Modulating Effects of UVC/PHA

**Figure 2:**
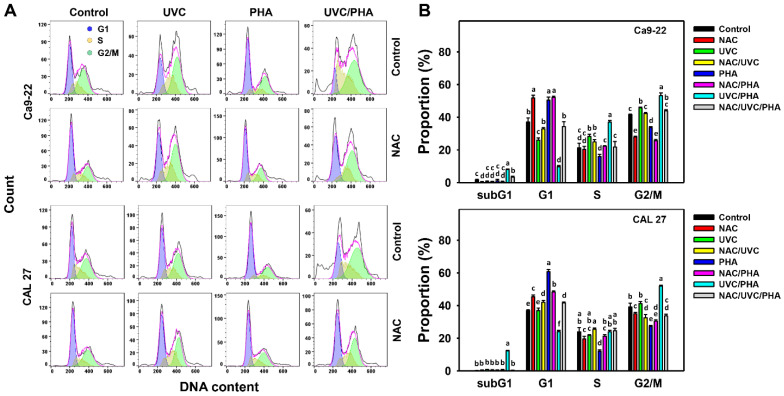
UVC/PHA-induced subG1 accumulation and G2/M arrest in oral cancer cells. In the presence or absence of NAC, oral cancer cells were grouped into four treatments, i.e., control, UVC, PHA, and UVC/PHA, for 24 h. The UVC and PHA treatment doses were 8 J/m^2^ and 0.8 μM, respectively. (**A**,**B**) Profile and data. For comparison, treatments labeled with different letters (a to e) produced significantly different results (*p* < 0.05). Data are presented as mean ± SD (*n* = 3 independent experiments, with each experiment involving 5000 gated cell counts).

The cell cycle profile (Figure 2A) showed that UVC/PHA increased subG1 (%) and G2/M (%) but decreased G1 (%) in oral cancer cells (Ca9-22 and CAL 27) compared to the control, UVC, and PHA treatments (Figure 2B). NAC reversed the UVC/PHA-induced cell cycle changes, suggesting that UVC/PHA exerted a more potent oxidative-stress-dependent effect on the cell cycle progression of oral cancer cells compared to the separate treatments.

### 3.3. Apoptosis-Modulating Effects of UVC/PHA

**Figure 3:**
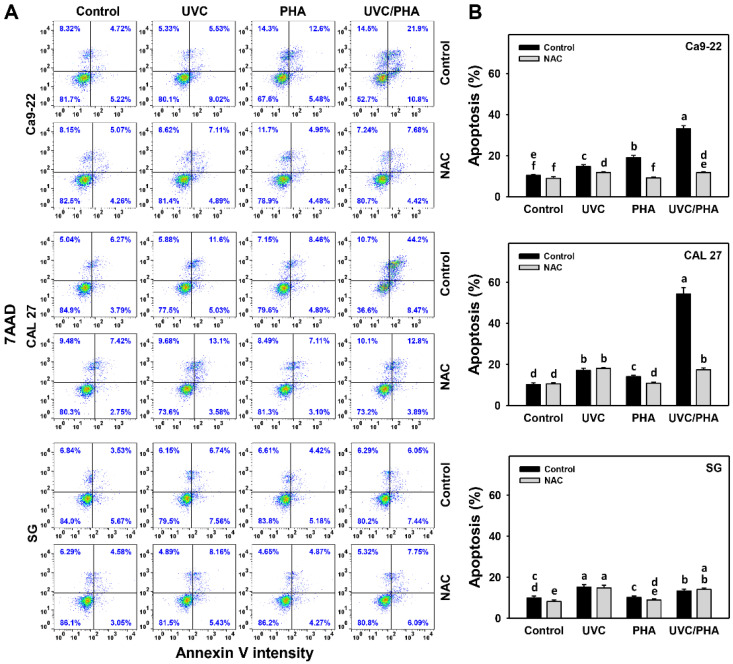
UVC/PHA-induced annexin-V-detected apoptosis in oral cancer cells. In the presence or absence of NAC, oral cancer and normal (SG) cells were grouped into four treatments, i.e., control, UVC, PHA, and UVC/PHA, for 24 h. The doses for the UVC and PHA treatments were 8 J/m^2^ and 0.8 μM, respectively. (**A**,**B**) Profile and data. Annexin-V-positive results (%) indicated apoptosis (%). For comparison, treatments labeled with different letters (a to f) produced significantly different results (*p* < 0.05). Data are presented as mean ± SD (*n* = 3 independent experiments, with each experiment involving 5000 gated cell counts).

The annexin V/7AAD profile (Figure 3A) showed that UVC/PHA induced a greater extent of apoptosis in oral cancer cells (%), as displayed by annexin V (+), than the control, UVC, and PHA treatments (Figure 3B). Moreover, UVC/PHA displayed a greater extent of annexin V (+)-indicated apoptosis (%) in oral cancer cells than in normal cells (SG) (Figure 3B). NAC reversed the UVC/PHA-induced annexin V changes, suggesting that UVC/PHA induced a greater extent of oxidative-stress-dependent apoptosis in oral cancer cells than the separate treatments.

**Figure 4:**
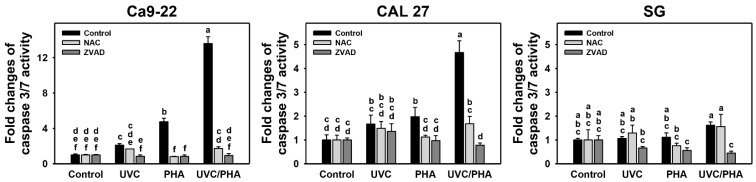
UVC/PHA-induced Cas 3/7 activity in oral cancer cells. In the presence or absence of NAC or ZVAD, oral cancer and normal (SG) cells were grouped into four treatments, i.e., control, UVC, PHA, and UVC/PHA, for 24 h. The doses of UVC and PHA treatments were 8 J/m^2^ and 0.8 μM, respectively. For comparison, treatments labeled with different letters (a to f) produced significantly different results (*p* < 0.05). Data are presented as mean ± SD (*n* = 3 independent experiments, with each experiment performed in three replications).

In addition to the annexin V/7AAD examination, the Cas 3/7 activity was also assessed (Figure 4). The oral cancer cells treated with UVC/PHA displayed a greater extent of Cas 3/7 activity than the control or those treated with UVC or PHA alone. Moreover, UVC/PHA induced higher Cas 3/7 activity in oral cancer cells than in normal cells (SG). NAC and ZVAD reversed the UVC/PHA-induced Cas 3/7 activity changes while showing mild changes in normal cells. Consequently, UVC/PHA induced greater oxidative-stress-dependent Cas 3/7 activation in oral cancer cells compared to the separate treatments.

### 3.4. ROS-Modulating Effects of UVC/PHA

**Figure 5:**
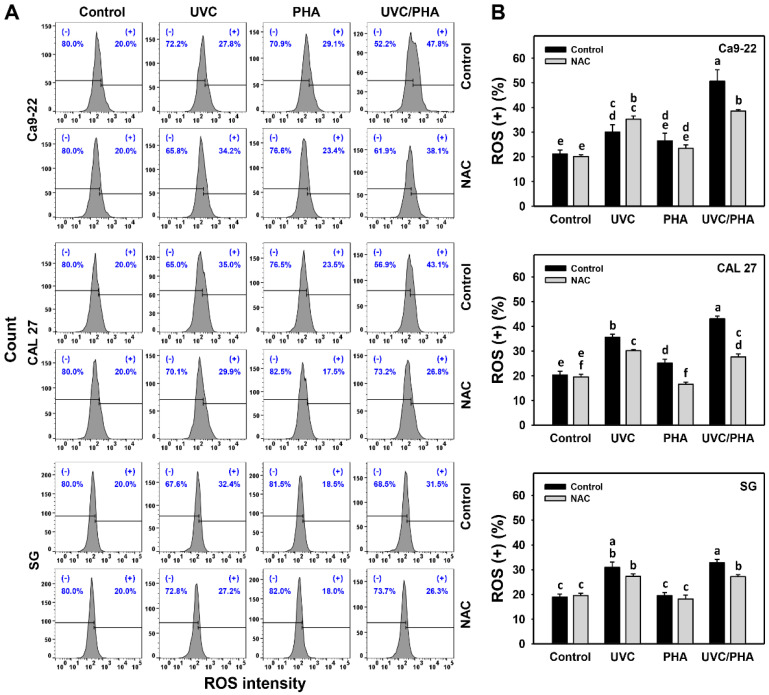
UVC/PHA-induced ROS in oral cancer cells. In the presence or absence of NAC, oral cancer and normal (SG) cells were grouped into four treatments, i.e., control, UVC, PHA, and UVC/PHA, for 24 h. The doses for the UVC and PHA treatments were 8 J/m^2^ and 0.8 μM, respectively. (**A**,**B**) Profile and data. (+) (%) indicates ROS-positive (%). For comparison, treatments labeled with different letters (a to f) produced significantly different results (*p* < 0.05). Data are presented as mean ± SD (*n* = 3 independent experiments, with each experiment involving 5000 gated cell counts).

The ROS profile (Figure 5A) showed that the oral cancer cells treated with UVC/PHA displayed a greater extent of ROS (+) (%) than the control or those treated with UVC or PHA (Figure 5B). Moreover, UVC/PHA induced a greater extent of ROS (+) (%) in oral cancer cells than in normal cells (SG) (Figure 5B). NAC reversed the UVC/PHA-induced ROS changes, suggesting that UVC/PHA exerted more potent oxidative stress on oral cancer cells than the separate treatments.

### 3.5. MitoSOX-Modulating Effects of UVC/PHA

**Figure 6:**
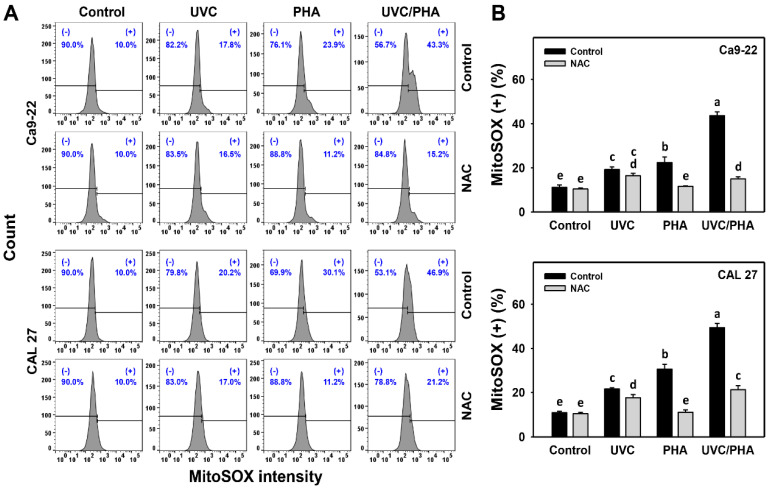
UVC/PHA-induced MitoSOX in oral cancer cells. In the presence or absence of NAC, oral cancer cells were grouped into four treatments, i.e., control, UVC, PHA, and UVC/PHA, for 24 h. The doses for the UVC and PHA treatments were 8 J/m^2^ and 0.8 μM, respectively. (**A**,**B**) Profile and data. (+) (%) indicates MitoSOX-positive (%). For comparison, treatments labeled with different letters (a to e) produced significantly different results (*p* < 0.05). Data are presented as mean ± SD (*n* = 3 independent experiments, with each experiment involving 5000 gated cell counts).

The MitoSOX profile (Figure 6A) showed that the oral cancer cells treated with UVC/PHA displayed a greater extent of MitoSOX (+) (%) than the control and those treated with UVC or PHA (Figure 6B). Moreover, NAC reversed the UVC/PHA-induced MitoSOX changes, suggesting that UVC/PHA exerted more potent oxidative stress on oral cancer cells than the separate treatments.

### 3.6. MitoMP-Modulating Effects of UVC/PHA

**Figure 7:**
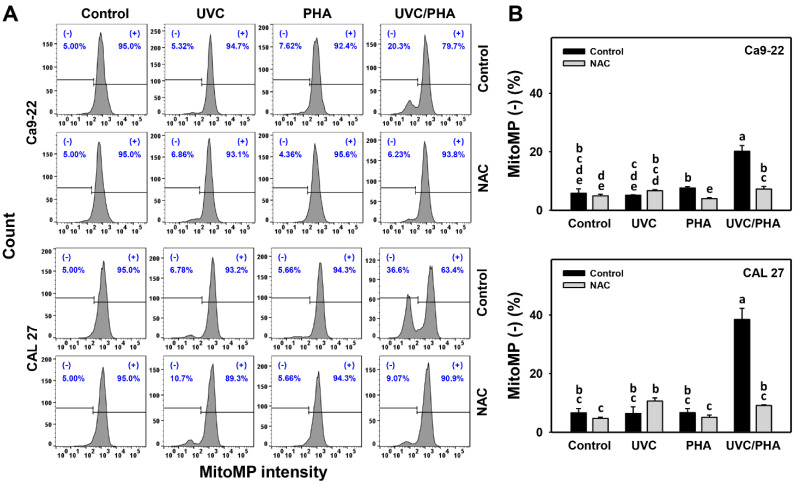
UVC/PHA-induced MitoMP depletion in oral cancer cells. In the presence or absence of NAC, oral cancer cells were grouped into four treatments, i.e., control, UVC, PHA, and UVC/PHA, for 24 h. The doses for the UVC and PHA treatments were 8 J/m^2^ and 0.8 μM, respectively. (**A**,**B**) Profile and data. (−) (%) indicates MitoMP-negative (%). For comparison, treatments labeled with different letters (a to e) produced significantly different results (*p* < 0.05). Data are presented as mean ± SD (*n* = 3 independent experiments, with each experiment involving 5000 gated cell counts).

The MitoMP profile (Figure 7A) showed that the oral cancer cells treated with UVC/PHA displayed a greater extent of MitoMP (−) (%) than the control and those treated with UVC or PHA (Figure 7B). Moreover, NAC reversed the UVC/PHA-induced MitoMP changes, suggesting that UVC/PHA exerted a more potent oxidative-stress-dependent effect on the MitoMP in oral cancer cells than the separate treatments.

### 3.7. DNA-Damage-Modulating Effects of UVC/PHA

**Figure 8:**
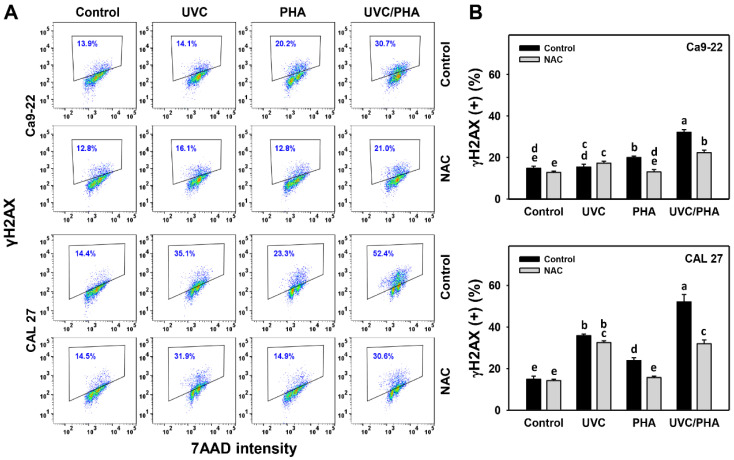
UVC/PHA-induced γH2AX in oral cancer cells. In the presence or absence of NAC, oral cancer cells were grouped into four treatments, i.e., control, UVC, PHA, and UVC/PHA, for 24 h. The doses for the UVC and PHA treatments were 8 J/m^2^ and 0.8 μM, respectively. (**A**,**B**) Profile and data. Percentages within the boxes indicate γH2AX-positive (%). For comparison, treatments labeled with different letters (a to e) produced significantly different results (*p* < 0.05). Data are presented as mean ± SD (*n* = 3 independent experiments, with each experiment involving 5000 gated cell counts).

**Figure 9:**
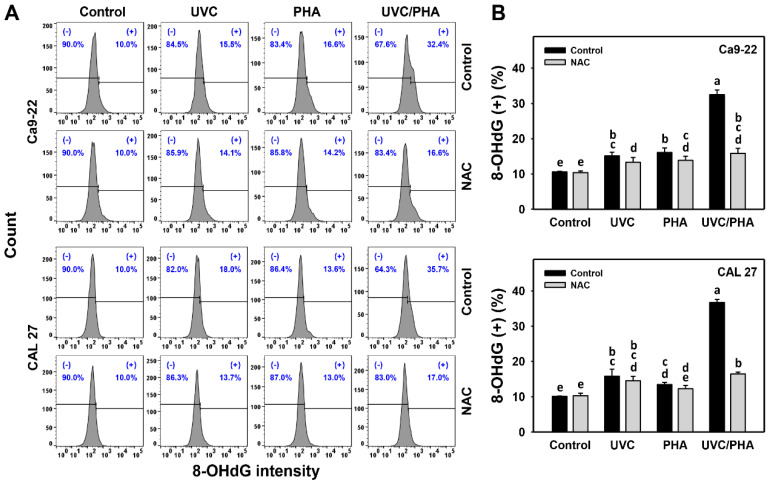
UVC/PHA-induced 8-OHdG in oral cancer cells. In the presence or absence of NAC, oral cancer cells were grouped into four treatments, i.e., control, UVC, PHA, and UVC/PHA, for 24 h. The doses for the UVC and PHA treatments were 8 J/m^2^ and 0.8 μM, respectively. (**A**,**B**) Profile and data. (+) (%) indicates 8-OHdG-positive (%). For comparison, treatments labeled with different letters (a to e) produced significantly different results (*p* < 0.05). Data are presented as mean ± SD (*n* = 3 independent experiments, with each experiment involving 5000 gated cell counts).

The γH2AX and 8-OHdG profiles (Figure 8A and Figure 9A) showed that the oral cancer cells treated with UVC/PHA displayed a greater extent of γH2AX and 8-OHdG (+) (%) than the control and those treated with UVC or PHA (Figure 8B and Figure 9B). Moreover, NAC reversed the UVC/PHA-induced γH2AX and 8-OHdG changes, suggesting that UVC/PHA exerted a more potent oxidative-stress-dependent DNA-damage effect on oral cancer cells than the separate treatments.

## 4. Discussion

PHA previously showed higher antiproliferation effects in oral cancer cells than in normal cells [27]. Currently, investigations into combined treatments involving PHA are rare. The present study confirmed that UVC/PHA caused a greater extent of antiproliferation in oral cancer than in normal cells. Several UVC/PHA-triggered responses to oral cancer cells were discussed.

Clinical drugs and natural products have been shown to enhance the UVC-induced antiproliferation effects on oral cancer cells. For example, a combined treatment of 10 μM cisplatin/10 J/m^2^ UVC inhibited proliferation in colon cancer cells to a greater extent than separate treatments [18]. Coral-derived sinularin [16] and brown-algae-derived fucoidan [20] showed more substantial antiproliferation effects against oral cancer cells when combined with UVC irradiation. Notably, combined treatments of UVC/sinularin [13] or UVC/fucoidan [17] showed higher antiproliferation effects in oral cancer than in normal cells. Similarly, PHA induced greater antiproliferation effects in oral cancer than normal cells [27], providing evidence of the preferential-antiproliferation property of PHA. Due to this preferential-antiproliferation property, PHA combined with UVC showed an enhancement of UVC- and PHA-induced antiproliferation effects in oral cancer cells compared to normal cells (Figure 1). This synergistic antiproliferation effect of UVC/PHA was reversed by NAC, revealing that oxidative stress is crucial in regulating these antiproliferation effects.

Several natural products, such as sinularin [13] and fucoidan [17], have demonstrated the ability to generate oxidative stress. UVC also exhibits the same capacity [30,45,46,47,48]. Consequently, combined treatments of these natural products and UVC are expected to produce stronger oxidative stress bursts than separate treatments. Similarly, UVC and PHA were confirmed to be oxidative-stress-inducing agents for oral cancer cells, as evidenced by ROS, MitoSOX, and MitoMP (Figure 5, Figure 6 and Figure 7). UVC/PHA produced a greater extent of oxidative stresses than the UVC or PHA treatments. This finding raises the possibility that UVC/PHA could provide a higher level of oxidative stress, leading to an increased antiproliferation effect against oral cancer cells. Moreover, UVC/PHA produced higher oxidative stress in oral cancer than in normal cells, confirming the preferential-antiproliferation property of UVC/PHA against oral cancer cells (Figure 1).

Since oxidative stress causes apoptosis [49] and DNA damage [50], these phenomena need to be examined. As expected, the UVC/PHA-triggered oxidative stress bursts may have affected oxidative-stress-associated responses, as shown by the upregulation of γH2AX- and 8-OHdG-type DNA damage and caspase 3/7-indicated apoptosis in oral cancer cells compared to the separate treatments (Figure 8, Figure 9 and Figure 4), respectively.

As mentioned above, UVC/PHA induced a greater extent of oxidative-stress-associated responses; therefore, oxidative stress dependence needs further assessment. By applying NAC, all these oxidative-stress-associated responses, such as abnormal cell cycle progression (subG1 increase and G2/M arrest), apoptosis, and DNA damage, were reversed. Notably, NAC had little inhibitory impact on the antiproliferative effect of UVC alone in oral cancer Ca9-22 or CAL 27 cells, depending on the experimental system (annexin V, ROS, or MitoMP). However, the measured impacts of PHA alone or UVC/PHA were suppressed by NAC, which was more effective for UVC/PHA. These results suggest that the combined treatment may have cooperatively enhanced the oxidative stress levels and responses in oral cancer cells compared to the separate treatments. Consequently, UVC/PHA exerted an oxidative-stress-associated mechanism of antiproliferation against oral cancer cells.

## 5. Conclusions

**Figure 10:**
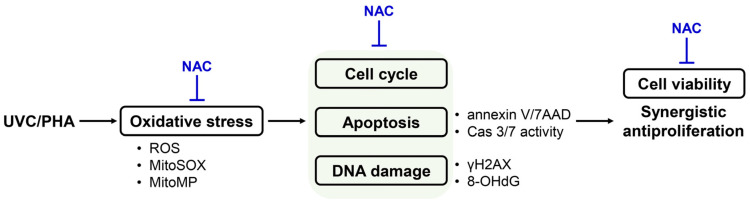
Synergistic molecular antiproliferation mechanism of UVC/PHA against oral cancer cells. In brief, the UVC/PHA combined treatment cooperatively generated more oxidative stress than separate treatments (UVC or PHA). Subsequently, the UVC/PHA-induced oxidative stress caused higher cell cycle disturbance, apoptosis, and DNA damage than UVC or PHA alone. Finally, these mechanisms of UVC/PHA action led to a synergistic antiproliferation effect against oral cancer cells. Moreover, NAC could suppress the UVC/PHA-associated changes, indicating that UVC/PHA induced an oxidative-stress-mediated synergistic antiproliferation effect against oral cancer cells.

Combined treatments benefit cancer therapy by reducing the drug dosages required but improving their effectiveness. This investigation confirmed that UVC/PHA exhibited an improved antiproliferation effect in oral cancer cells compared to UVC or PHA alone. UVC/PHA also reduced the level of proliferation and increased the extent of apoptosis, caspase 3/7 activation, and ROS generation in oral cancer compared to normal cells. In addition to ROS generation, UVC/PHA caused more mitochondrial dysfunction, including MitoSOX generation and MitoMP depletion, in oral cancer cells compared to UVC or PHA alone. NAC reversed these mechanisms triggered by UVC/PHA. Consequently, the role of oxidative stress in regulating these UVC/PHA-induced changes was validated. The synergistic molecular antiproliferation mechanism of UVC/PHA against oral cancer cells is illustrated in Figure 10. The present study investigated the combined treatment of UVC/PHA, which demonstrated an enhanced antiproliferation effect against oral cancer cells and a lack of harmful effects on normal cells, thus providing a potential oral cancer treatment.

## Data Availability

Data are contained within the article.

## Supplementary Materials

The following supporting information can be downloaded at: https://www.mdpi.com/article/10.3390/antiox11112227/s1, Table S1: The synergy determinations (α values) for all tested combined treatments with different UVC and/or PHA doses in oral cancer and normal cells.
