# Supplementary material for: Combined Treatment (Ultraviolet-C/Physapruin A) Enhances Antiproliferation and Oxidative-Stress-Associated Mechanism in Oral Cancer Cells
Source: Antioxidants (Basel). 2022 Nov 11;11(11):2227. doi: 10.3390/antiox11112227 (PMC9686797; doi:10.3390/antiox11112227)
Supplement: Supplementary file 1 [file antioxidants-11-02227-s001.zip › antioxidants-1962535-supplementary.pdf]

Table S1. Synergy values (α) for all combined treatments (UVC/PHA) to oral cancer (Ca9-22 and CAL 27) and normal (SG) cells

**Treatment**		**PHA (μM)**			
		**0.4**	**0.6**	**0.8**	
**UVC (J/m^2)**	**8**	1.06 ± 0.06	1.11 ± 0.07	1.15 ± 0.06	**Ca9-22**
	**10**	1.14 ± 0.06	1.13 ± 0.06	1.23 ± 0.10	
	**8**	1.17 ± 0.09	1.28 ± 0.01	1.46 ± 0.20	**CAL 27**
	**10**	1.06 ± 0.05	1.11 ± 0.06	1.25 ± 0.21	
	**8**	1.05 ± 0.00	1.02 ± 0.07	0.99 ± 0.06	**SG**
	**10**	1.09 ± 0.08	1.12 ± 0.04	1.09 ± 0.02	

Cells were irradiated with UVC and incubated in control and PHA (0.4, 0.6, and 0.8 μM) for 24 h. Finally, the cell viabilities were determined by ATP assay and the synergy values (α) were calculated as described in Materials and Methods. Data, mean±SD (n = 3).
